# Reducing molecular simulation time for AFM images based on super-resolution methods

**DOI:** 10.3762/bjnano.12.61

**Published:** 2021-07-29

**Authors:** Zhipeng Dou, Jianqiang Qian, Yingzi Li, Rui Lin, Jianhai Wang, Peng Cheng, Zeyu Xu

**Affiliations:** 1 School of Physics, Beihang University, Beijing 100083, China

**Keywords:** atomic force microscopy, Bayesian compressed sensing, convolutional neural network, molecular dynamics simulation, super resolution

## Abstract

Atomic force microscopy (AFM) has been an important tool for nanoscale imaging and characterization with atomic and subatomic resolution. Theoretical investigations are getting highly important for the interpretation of AFM images. Researchers have used molecular simulation to examine the AFM imaging mechanism. With a recent flurry of researches applying machine learning to AFM, AFM images obtained from molecular simulation have also been used as training data. However, the simulation is incredibly time consuming. In this paper, we apply super-resolution methods, including compressed sensing and deep learning methods, to reconstruct simulated images and to reduce simulation time. Several molecular simulation energy maps under different conditions are presented to demonstrate the performance of reconstruction algorithms. Through the analysis of reconstructed results, we find that both presented algorithms could complete the reconstruction with good quality and greatly reduce simulation time. Moreover, the super-resolution methods can be used to speed up the generation of training data and vary simulation resolution for AFM machine learning.

## Introduction

Atomic force microscopy methods are key tools for nanoscale imaging and characterization with unparalleled resolution [1]. The first atomic-resolution image by AFM of the (001) surface of NaCl was reported in ultrahigh vacuum [2]. Later, in noncontact mode, the reconstructed silicon (111)-(7×7) surface was imaged with 6 Å lateral and 0.1 Å vertical resolution [3]. Then, functionalizing the tip with closed-shell molecules and using a qPlus force sensor enabled the imaging of the internal structure of the molecules [4–7], resolving features of weak-bonded molecules [8–12], and measurement of bond-order relations [13–15]. The interpretation of AFM images, however, is often complicated. Theoretical investigations are getting highly important for the analysis of AFM images. Molecular simulation is a useful tool to study atomic-scale interactions. It has been used to examine the AFM imaging mechanism, study the factors affecting resolution [16–22], and establish an appropriate simulation methodology for the explanation of complex imaging mechanism in liquids [23–25]. Besides, there has been a recent flurry of researches applying machine learning to AFM, including predicting the molecular structure [26], recognizing and classifying surface features [27–29], and fast scanning [30–31]. The main problem in training models of machine learning is providing sufficiently labeled training data. High-resolution AFM experiments are time consuming and experimental data are, a priori, unlabeled, which would render the direct training on experimental data impractical. Researchers have used simulated AFM images as training data, in which correct labels are known a priori, and then the trained model correctly generalizes to real AFM scans [26,32–33]. Molecular simulation methods are effective for AFM image analysis and training data generation; however, it takes a lot of time to get a complete image of a sample surface. Molecular simulation imaging generally simulates the interaction of tip atoms in a point-to-point scan of a sample. An image contains hundreds of pixels and each pixel needs to be simulated once. The most direct way to reduce simulation time is to decrease the number of calculated points and reconstruct the whole image from undersampling points.

For the above problem, we could use super-resolution methods [34–35], which refers to a classical concept in computer vision. Super-resolution methods could be used to reconstruct a high-resolution image from a low-resolution image. There are a variety of methods in the field of super resolution. Compressed sensing (CS) and deep learning methods are two typical methods with excellent imaging performance [36], which use the principle of signal sparsity and the learning ability of deep neural networks to achieve super-resolution tasks. The CS has the possibility of recovering the data almost perfectly from undersampled information [37], which is widely used in AFM imaging to reduce sampling time [38–43]. The CS algorithm is mainly divided into three parts including the construction of the measurement matrix, signal sparsity, and image reconstruction. In some sense, taking a random measurement matrix is an optimal strategy for the acquirement of sparse signals [44]. The choice of the reconstruction algorithm is essential for CS. The Bayesian compressed sensing (BCS) can achieve a better recovery performance due to its high degree of sparsity and the ability to estimate the posterior distribution of the reconstructed signal [45–48], even if the signal is not sparse. In addition to using the signal sparsity, learning-based super-resolution reconstruction methods are also focused issues and trends in research. The super-resolution convolutional neural network (SRCNN) is the first neural network model for super-resolution reconstruction tasks [35]. The SRCNN consists of a three-layer convolutional neural network which directly learns an end-to-end mapping between low- and high-resolution images, making the reconstructed image as close to the original image as possible. Generally, increasing the network depth could improve the reconstruction accuracy. With the development of deep learning in super-resolution methods many other models have been proposed, such as VGG [49], Res Net [50], GAN [51], and VDSR [52]. The application of deep learning in super-resolution methods has been increasing in recent years, and it is also used in AFM to speed up imaging acquisition [31,53]. The abovementioned reconstruction algorithms are mostly aimed at real-scanned images, but they could have better applications in molecular simulation to speed up simulations.

In this paper, BCS and SRCNN methods are used to reconstruct the molecular simulation images to reduce simulation time. The energy maps of the interaction between several tips and samples under different conditions are simulated in dynamic and quasi-static modes. The molecular dynamics simulation details and main steps of reconstruction algorithms are presented. Then, several reconstruction results are conducted to compare the performance of these two algorithms. We find that both presented algorithms can complete the reconstruction with good quality and reduce the simulation time. In addition, we could use these two algorithms to speed up the generation of training data for AFM machine learning.

## Methods

### Molecular dynamics simulation

**Figure 1:**
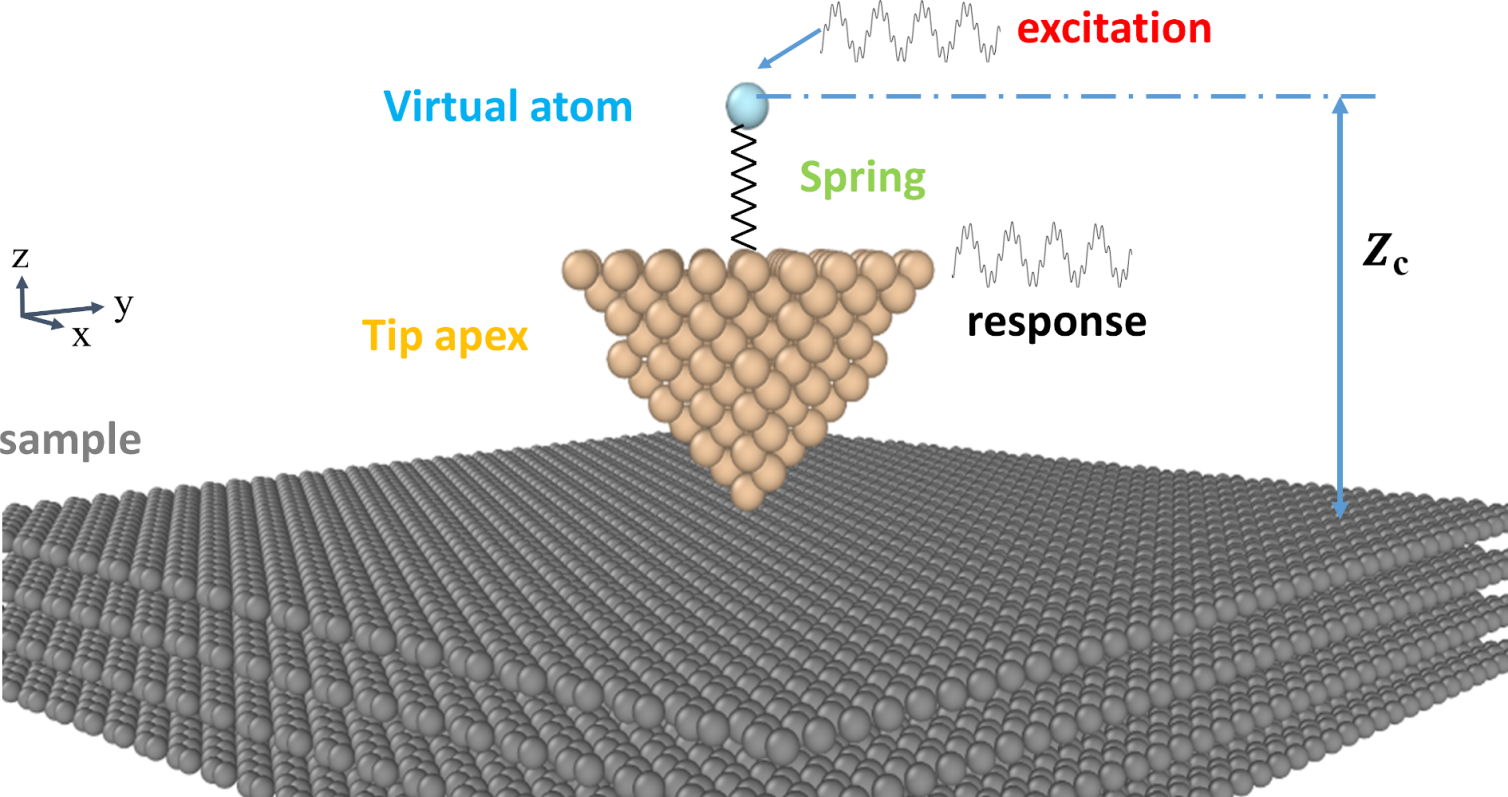
Snapshot of the AM mode molecular dynamics simulation. *Z*_c_ is the distance between the virtual atom at rest and the sample surface. The dynamic response of the tip is determined by the excitation of the virtual atom and tip–sample interactions. In the simulation the tip is parallel to the sample surface and the scanning is done in raster scan path.

To test the effectiveness of the reconstruction algorithms we perform molecular dynamics simulations of AFM imaging in different conditions. The dynamic process (AM mode) and quasi-static process (the relative position of tip–sample remains unchanged in the simulation) are separately carried out. The simulation protocol for computing tip–sample interactions is similar to our previous work [54]. In the simulations, the tip consists of silicon atoms and it changes among different shapes, including cone, hemisphere, and single silicon atom. The conical tip height is 13 Å, the opening angle is 70°, and the hemispherical tip radius is 16 Å. We calculate the surface energy maps with a four-layer graphite and gold samples. The dimensions of the graphite and gold substrates are 9 × 9 × 1.1 and 9 × 9 × 0.4 nm^3^, respectively. All simulations are performed under equal height conditions. The schematic of the AM mode simulation model with conical tip apex is illustrated in Figure 1. The bottom layer atoms of the substrate are fixed to keep the sample stable. For the graphite substrate, the carbon–carbon interactions within each graphene layer are described by the AIREBO potential [55]. The Lennard–Jones (LJ) potential is used to describe the interaction between the graphene layers and the tip substrate. The LJ parameters for C–C are ε_C–C_ = 2.84 meV, σ_C–C_ = 0.34 nm and for Si–C the parameters are ε_Si–C_ = 8.909 meV, σ_Si–C_ = 0.3326 nm (ε is the depth of the potential well, σ is the finite distance). The cut-off distance for the C–C interaction is 1.19 nm. The cut-off distance for the Si–C interaction is changed to observe the impact on the simulation. Increasing the cut-off distance increases the number of atoms in the tip–sample interaction but the result is more accurate. For the gold substrate, the interaction between Au atoms is calculated by the embedded atom method (EAM) potential [56]. The LJ potential is employed to calculate the interaction between Si and Au (ε_Si–Au_ = 5.4297 meV, σ_Si–Au_ = 0.33801 nm). Due to the relatively large size of the substrate compared to the tip and a small cut-off distance, the effect of the boundary could be ignored. The NVT ensemble (the number of atoms, volume, and temperature are conserved) is employed to the free atoms in the system. All simulations are performed using the LAMMPS simulation software with a time step of 1 fs.

In AM mode, a virtual atom is added above the tip apex and they are connected with a spring in the *z*-direction. The virtual atom is excited by a sinusoidal signal, mimicking the acoustic excitation of AM-AFM. The excited frequency is adjusted by the spring stiffness. A damping force is applied on the tip to ensure the correct damping of the tip oscillation. In the simulation, the initial distance between the virtual atom and the sample surface *Z*_c_ remains unchanged at a suitable distance. Then we employ the raster scanning method to construct the average interplay energy map of the sample surface in a calculation period. In the scan, the tip is parallel to the sample surface in steps of 0.1 Å. After scanning 51 points, the tip turns to the next line until all are scanned. Then, the average energy map of the tip–sample interaction is obtained. In a quasi-static simulation, the tip is not excited and the conical tip is changed to a hemisphere and a single atom. The tip is typically scanned parallel to the surface at a fixed distance to the molecule being investigated. The raster scanning method is also applied for the quasi-static mode with a size of 101 × 101.

### Bayesian compressed sensing

For linear systems, the signal acquisition process can be simulated by a linear equation. In the molecular simulation of an AFM image, every pixel of the image is calculated independently. A *n* × *n* simulated image can be seen as a 2D matrix and all rows of the matrix can form a vector *x* ∈ R*^N^* (*N* = *n* × *n*). The entire signal acquisition process can be described as

**Figure 2:**
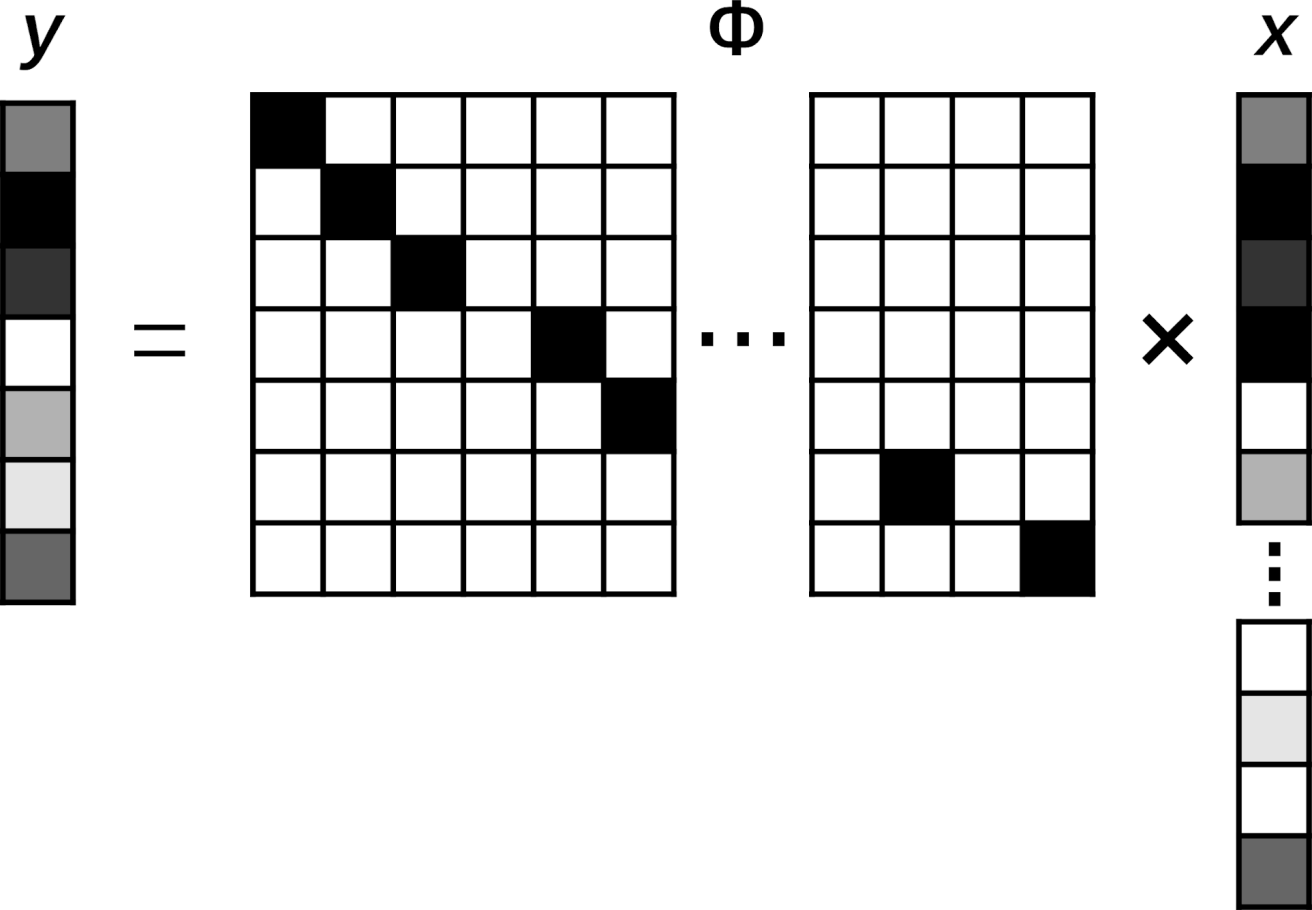
Mathematical model of compressed-sensing AFM imaging. The image obtained by AFM can be regarded as a two-dimensional matrix, and each pixel of the image is an element of the matrix. The imaging process can be regarded as collecting the element of the vector *x*, the Φ is the measurement matrix, and *y* is the measurement result.

[1] y=Φx,

where *x* is the true value of the original signal, and *y* ∈ R*^M^* is the measured value of the original signal. The matrix Φ ∈ R*^M^*^×^*^M^* represents the linear measurement matrix that obtains the *M*-dimensional measurement results in the *N*-dimensional space, as shown in Figure 2. If *M* < *N*, it means that the linear observation system is underdetermined and we could use the sparsity of the signal to complete the reconstruction.

Signal sparsity means that there are only few non-zero elements contained in the signal. The simulated AFM images are usually not sparse but will show sparsity after the discrete cosine transform (DCT). Supposing that the DCT operation is Ψ, Equation 1 is rewritten as

[2] y=Φx=ΦΨs=θs,

where *y* ∈ R*^M^* is a compressed sampling signal for the original signal *x* ∈ R*^N^*, and its dimension is smaller than the original signal (*M* < *N*). The original signal *x* can be represented by the coefficient vector *s* of *K* sparse under the transform domain of the sparse basis Ψ. The product of the measurement matrix and the sparse transform matrix is represented by θ = ΦΨ. Such a CS framework can recover linear equations with unique sparse solutions.

The designs of the measurement matrix and reconstruction algorithms are important for CS algorithms. We set the measurement matrix to a random matrix for an idealized CS application and a fast reconstruction algorithm based on BCS is used to reconstruct the images. Since the measurement is random, the conditional probability density function of the measurement can be described by a zero-mean Gaussian distribution with variance σ^2^. The CS reconstruction problem is converted into a linear regression problem and the parameter *s* can be obtained by the maximum likelihood method. The details of the BCS reconstruction algorithm in AFM are given in our previous work [47].

[3] $$p\left(y|s,\sigma^{2}\right)={\left(2\pi \right)}^{-N/2}\sigma^{-N}\exp\left\{-\frac{\left\|y-\theta s\right\|}{2\sigma^{2}}\right\}$$

**Figure 3:**
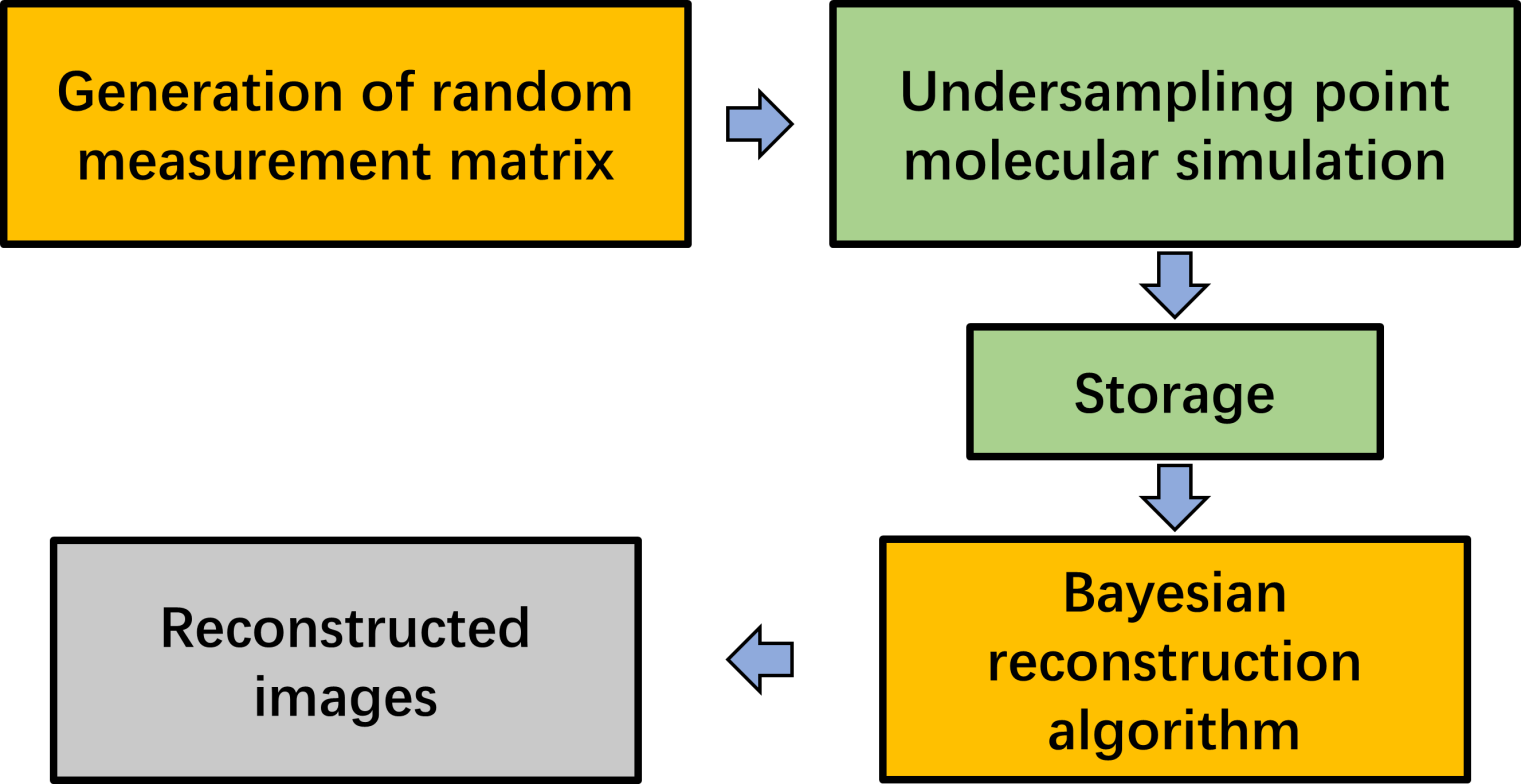
The block diagram of fast molecular simulation method for AFM imaging based on Bayesian compressed sensing. First, the random measurement matrix is obtained, then the pixel value is simulated according to random matrix positions, and finally the BCS method is used to reconstruct the complete image.

The whole operation process is shown in Figure 3. First, we need to generate a random measurement matrix according to the size of the final image and the number of compressed sampling points. For molecular simulation, the measurement matrix needs to be transformed into the required sampling position. Then, the interaction energy between tip and sample is calculated according to the sampling position. After that, we would get the undersampling images of the surface. Finally, the BCS reconstruction algorithm is used to reconstruct the whole images.

### Super-resolution convolutional neural network

SRCNN is a representative method for deep learning super-resolution techniques and consists of three layers. This method uses bicubic interpolation as the preprocessing step and, then, extracts overlapping image blocks as high-dimensional vectors with the same dimension as the feature image through convolution. After that, the high-dimensional vectors are nonlinearly mapped to each other and comprise another set of feature maps. Finally, they are aggregated into patches to obtain a reconstructed high-resolution image. A SRCNN is constructed to obtain a super-resolution image from a low-resolution simulated image [35]. Filters of spatial sizes of 9 × 9, 5 × 5, and 5 × 5 were used.

**Figure 4:**
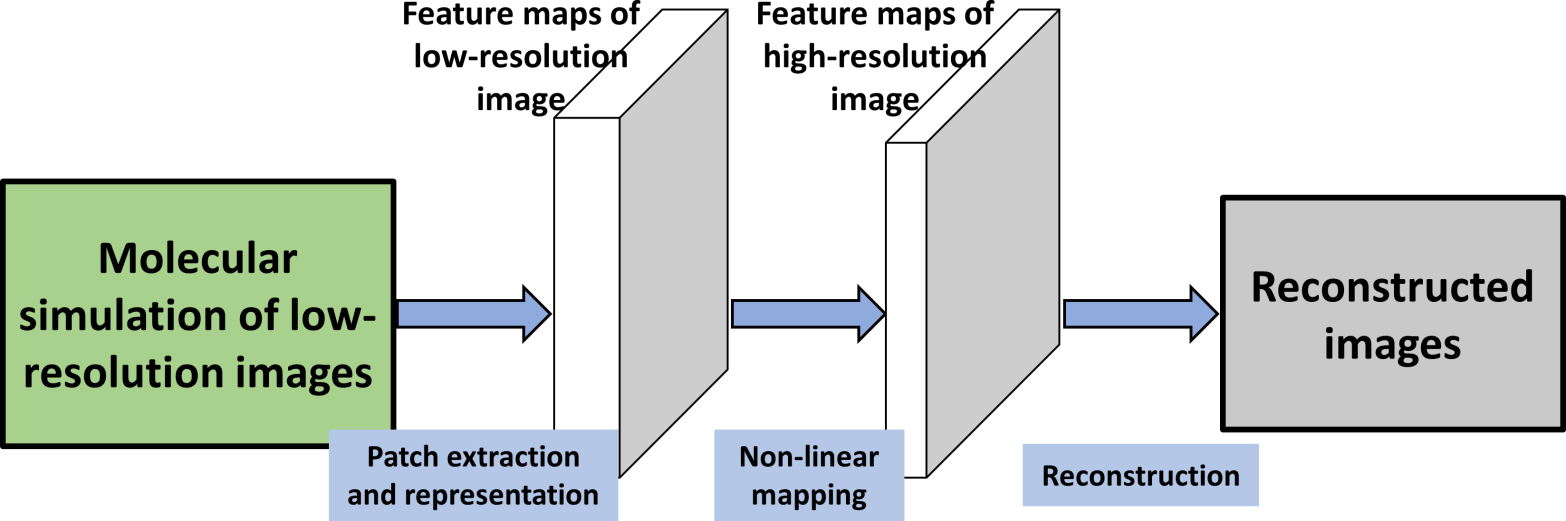
The flow chart of the proposed SRCNN reconstruction method. First, the low-resolution simulated image is obtained, then the SRCNN method is used to reconstruct the high-resolution image. The SRCNN consists of three layers to implement feature extraction, non-linear mapping, and reconstruction, respectively.

The flow chart of the proposed SRCNN reconstruction method is shown in Figure 4. First, we use molecular dynamics simulation to obtain energy maps of the tip–sample interaction with low-resolution. Different from the full image simulation, the scanning step size of a low-resolution image is increased by two or three times. The simulated points of the AFM images are reduced to 1/4 and 1/9, respectively. Then the low-resolution image is expanded to the desired size through bicubic interpolation, which is denoted as *X*. The first layer is operated to extract the image feature of *X* as an operation *F*_1_:

[4] $$F_{1}\left(X\right)=\max\left(0,W_{1}*X+B_{1}\right),$$

where *W*_1_ represents 64 types of convolution kernels with the size of 9 × 9, and *B*_1_ represents the biases. The first layer extracts a 64-dimensional feature for each patch. Furthermore, the non-linear mapping is on a 5 × 5 “patch” of the feature map as follows:

[5] $$F_{2}\left(X\right)=\max\left(0,W_{2}*F_{1}\left(X\right)+B_{2}\right),$$

where *W*_2_ and *B*_2_ represent 32 types of convolution kernels with the size of 5 × 5 and the biases, respectively. The final super-resolution image is generated with the reconstruction layer as follows:

[6] $$F_{3}\left(X\right)=W_{3}*F_{2}\left(X\right)+B_{3},$$

where *W*_3_ and *B*_3_ represent a linear filter with the size of 5 × 5 and the bias, respectively. The network parameters Θ = {*W*_1_, *B*_1, _*W*_2_, *B*_2_, *W*_3_, *B*_3_} are trained to construct the end-to-end mapping between low- and high-resolution images. Given a set of high-resolution images {*Y*_i_} and their corresponding low-resolution images {*X*_i_}, the mean squared error is used as the loss function:

[7] $$L\left(\Theta \right)=\frac{1}{n}{\sum_{i=1}^{n}\left\|F\left(X_{i};\Theta \right)-Y_{i}\right\|}^{2},$$

where *n* is the number of training samples. The parameters are updated with the gradient descent as

[8] $$\Theta_{i+1}=\Theta_{i}+\Delta_{i+1},\;\Delta_{i+1}=\Delta_{i}+\eta \frac{\partial L}{\partial \Delta_{i}},$$

where η denotes the learning rate.

## Results and Discussion

### Molecular dynamics simulation results

**Figure 5:**
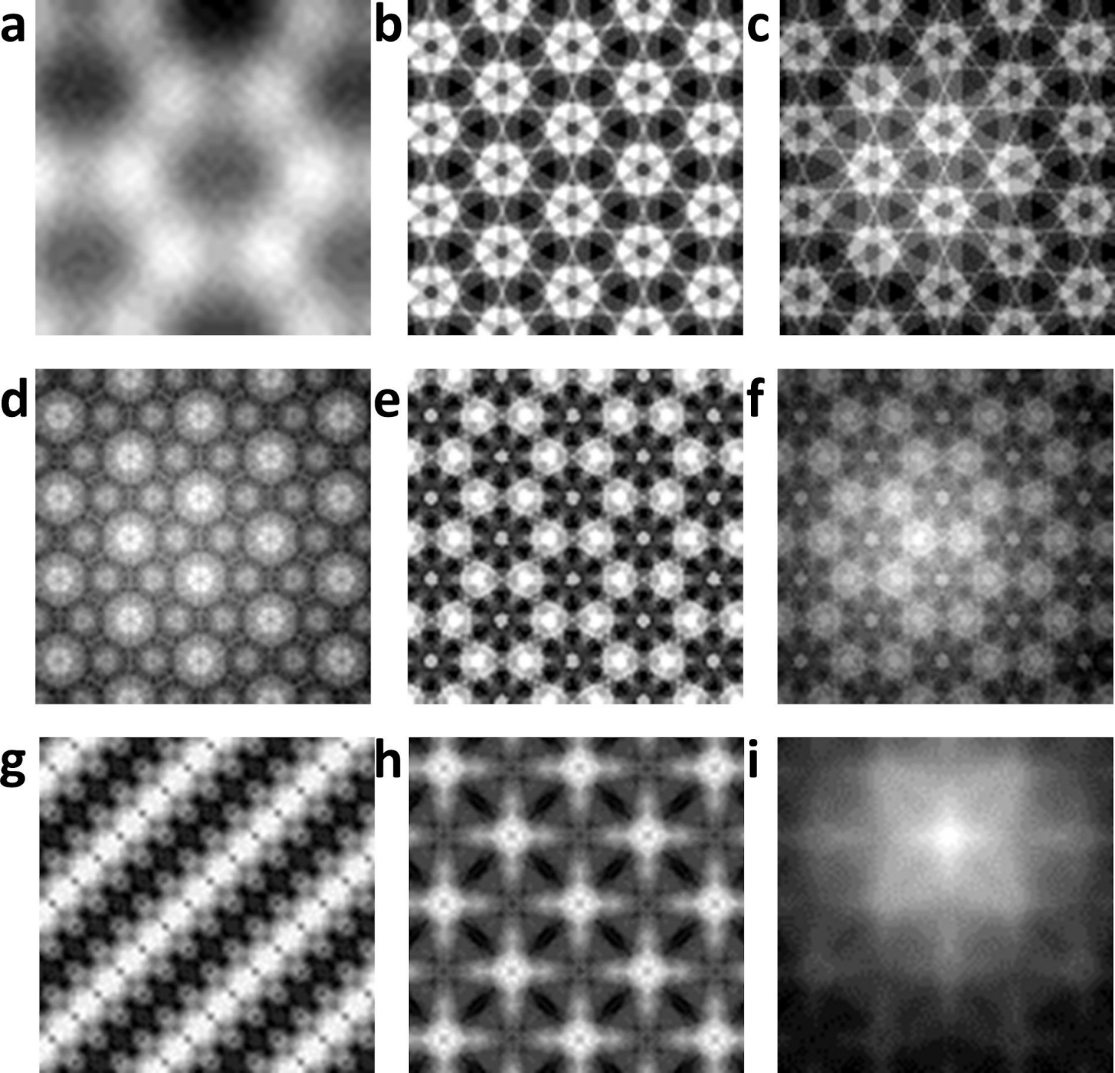
The interaction energy maps of graphite and gold samples obtained by molecular dynamics simulation. The size of the first image is 51 × 51 and the other images are 101 × 101. (a) The conical tip interacts with the graphite sample in AM mode. (b–f) The single Si atom tip interacts with the graphite sample at 7 Å. In (d) the cut-off distance of the tip–sample increases by 2 Å. In (e) the tip atom drops by 2 Å. (g) The hemispherical tip interacts with the gold sample. (h–i) The single Si atom tip interacts with the gold sample. In the simulations on the far-right column (c,f,i) we removed one atom from the sample surface.

Molecular dynamics simulation is used to obtain the energy maps of graphite and gold samples under different conditions for reconstruction, as shown in Figure 5. The first two rows represent the graphite sample and the last row is the gold sample. Figure 5a represents the interaction between the conical tip and the graphite sample in AM mode, as described in Figure 1. In this situation, the tip atoms intermittently interact with the sample in oscillation periods. The size effect of the tip apex is not obvious and the energy map is mainly affected by the height of the sample atoms. In the AM mode, we need 5000 time steps to keep the tip in a stable vibration to, then, calculate the average interaction energy. The inter-layer and intra-layer C–C interactions in the graphite sample are different and need to be calculated separately. In this simulation, one pixel would take about six minutes; therefore, a 51 × 51 image would take approximately ten days to be simulated. The other images of Figure 5 are simulated under quasi-static conditions. The tip remains stationary at an appropriate position on the sample surface and there is no need to spend time making the tip vibrate. Images of sizes 101 × 101 for graphite and gold samples with 2000 time steps would cost 56 and two hours, respectively. For more complex sample structures and potentials, especially electronic structures, may take more time. In quasi-static simulations, we could find that the tip shapes have a great influence on the results and are convolved into the energy maps. Different simulation conditions would also affect the final imaging results, such as tip–sample height and cut-off distance, for example. We generally need to perform multiple simulations to determine the appropriate simulation conditions. However, the number of calculation points required to form a whole image is huge, so it is necessary to shorten the simulation time by reducing the number of sampling points.

### Image reconstruction

**Figure 6:**
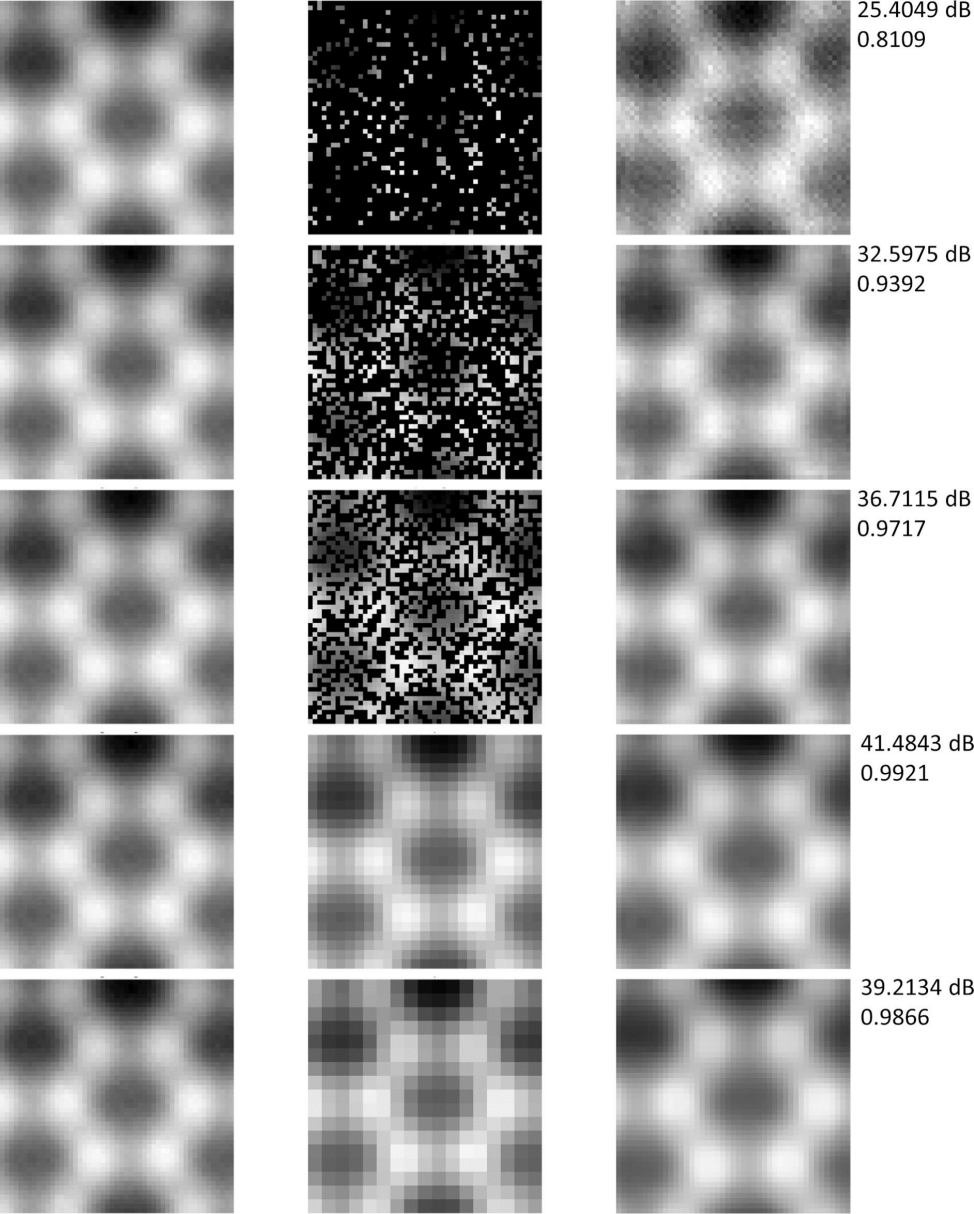
The reconstructed results of Figure 5a with size of 51 × 51. The first column is the original image, the second column is the undersampling image (or low-resolution image), and the last column is the reconstructed image. PSNR and SSIM are indicated on the right side of each image. The first three lines are the BCS results with under-sampling rates of 0.11, 0.3, and 0.5, respectively. The last two lines are the SRCNN results with scale factors of 2× and 3×, respectively.

In this part, we compare the performance of BCS and SRCNN algorithms for the simulated AFM images. Figure 5a with a size of 51 × 51 is used to test the impact of the sampling points on the reconstructed results, as shown in Figure 6. Generally, an undersampling rate of 0.5 could ensure a better reconstruction effect for BCS [47]. This image contains 2601 pixels and the undersampling rates of 0.11 (298 pixels), 0.30 (784 pixels), and 0.50 (1300 pixels) are chosen to reconstruct the complete image. For SRCNN, we set the scale factors to 2× (25 × 25) and 3× (17 × 17), respectively, to construct super-resolution images. The peak signal to noise ratio (PSNR, expressed as the logarithmic decibel scale) and structural similarity (SSIM, value range is [0,1]) are used to evaluate the quality of the reconstructed image. The PSNR is usually used to determine the image quality loss after image compression, denoising, and reconstruction. The SSIM is a measure of the similarity between two images. From Figure 6 we could find that with an increase in the undersampling rate, the reconstruction quality gradually improves. The reconstructed quality of the BCS method is mainly related to the undersampling rate. When the undersampling rate is higher than the sparsity of the image, the reconstructed quality hardly changes [47]. An undersampling rate of 0.5 could guarantee a good reconstruction quality. This means that we only need to calculate half of the points to reconstruct a complete simulated image by the BCS method. For the SRCNN method, both 2× and 3× scale factors could reconstruct the image well and the reconstructed quality is better than that of BCS. These results indicate that we could use SRCNN to reduce the simulation time to 1/4 and 1/9 of the original time, respectively. Overall, it shows that for simulated images with less details, two algorithms have a better reconstruction performance. However, BCS requires more sampling points to ensure reconstruction quality. Besides, the time spent on the reconstruction algorithm is negligible relative to the molecular dynamics simulation.

**Figure 7:**
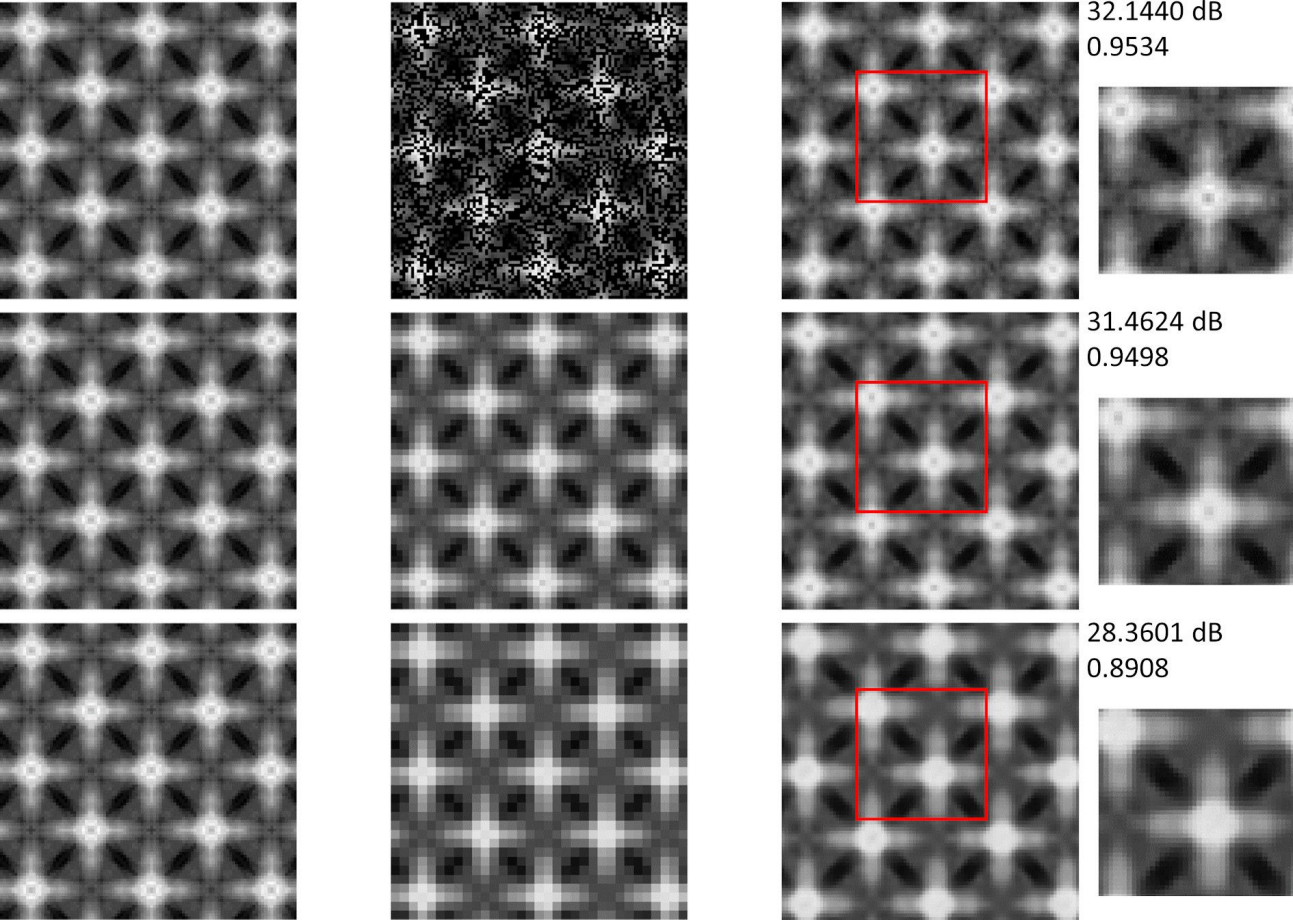
The reconstructed results of Figure 5h with size of 101 × 101. The first line is the result of the BCS algorithm with an undersampling rate of 0.5. The last two lines are the results of the SRCNN algorithm with scale factors of 2× and 3×, respectively. The small images on the right show the details of the reconstructed image in the red box.

The other images are reconstructed to compare the differences between two reconstruction algorithms. Figure 7 presents the reconstructed results of Figure 5h, which has more details and a larger size of 101 × 101. The undersampling rate of BCS is 0.5 and the scale factors of SRCNN are 2× and 3×. From the final reconstructed images we could find that for the image with more details both algorithms could complete the reconstruction. However, the quality of the image is reduced compared to the previous image. The PSNR and SSIM values of the first two lines in Figure 7 have little difference, but we could find from the detailed images on the right that the reconstructed quality of BCS is better than that of SRCNN and the details of the images are well reconstructed.

**Figure 8:**
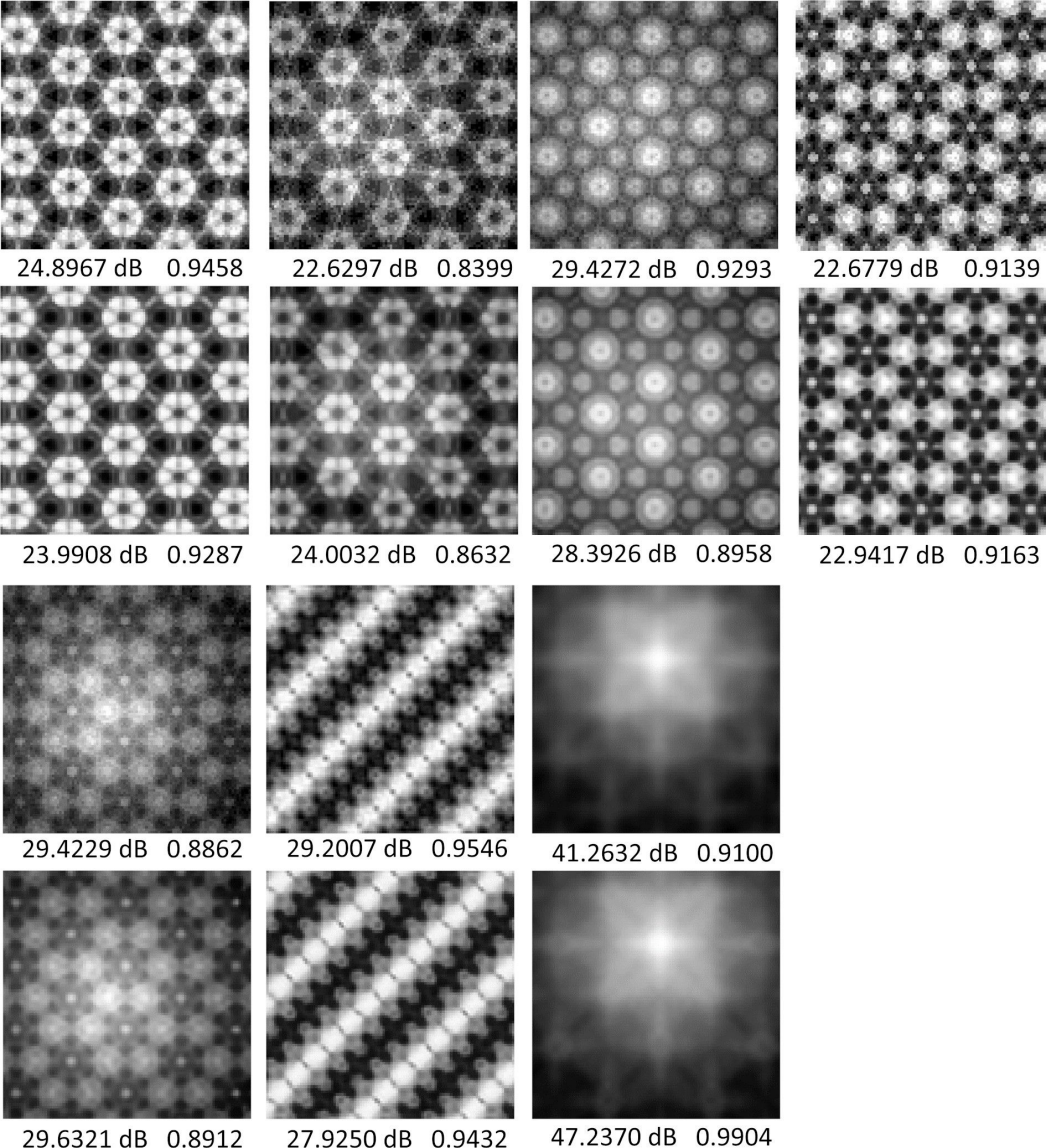
Reconstructed results of the other seven images. The upper image of each group shows the result of the BCS reconstruction with an undersampling rate of 0.5, and the lower image represents the SRCNN reconstruction with a scale factor of 2×. PSNR and SSIM are indicated below each image.

Other results are shown in Figure 8. Overall, both presented algorithms can complete the reconstruction. For images with less details, both algorithms can recover the simulated image from undersampled data with a high quality. The SRCNN method needs less simulated points and can reduce the simulation time to 1/9. However, for images with more details, SRCNN has poor quality on detail reconstruction. The BCS method could reconstruct the details with an undersampling rate of 0.5, which means that it could save half of the simulation time. Besides, more sampling points can ensure better reconstruction quality.

## Conclusion

In this paper, BCS and SRCNN methods are applied to reconstruct the molecular simulation images to reduce the simulation time. Several energy maps of graphite and gold samples under different conditions are simulated for image reconstruction. Both presented algorithms can reduce the time of molecular dynamics simulation and complete the reconstruction with good quality. Through undersampling and low-resolution mapping we could reduce the number of calculated points by 1/2 and 1/9, which means that the BCS method can reduce the simulation time by one half while the SRCNN method can reduce the simulation time to 1/9. By comparing the reconstruction results with different details, it can be found that SRCNN has better reconstruction quality when there are fewer details and BCS is better for detail reconstruction. For a preliminary simulation, we could choose the SRCNN method to achieve faster simulation and have a preliminary judgment on the final simulated results. If pursuing quality, the BCS method could be used to achieve a balance between time and quality while more sampling points can ensure better reconstruction quality. Besides, researchers typically use nearest-neighbor interpolation for upscaling the training images for the scale invariance in machine learning. The super-resolution methods could also be applied to vary simulation resolution, which is more accurate. In conclusion, these two methods can be used to speed up molecular simulation imaging and the generation of training data for AFM machine learning.
